# Forensic characteristics of 325 firearm-related autopsy cases in Bursa, Türkiye: a five-year retrospective study

**DOI:** 10.3389/fmed.2026.1865411

**Published:** 2026-06-04

**Authors:** Zeynep Doğan, Samet Kiyak

**Affiliations:** 1 Presidency of the Council of Forensic Medicine, Ministry of Justice of the Republic of Türkiye, Istanbul, Türkiye; 2 Department of Forensic Medicine, Faculty of Medicine, Balıkesir University, Balıkesir, Türkiye

**Keywords:** autopsy, firearm injury, forensic medicine, injury pattern, shooting distance, toxicology

## Abstract

**Background:**

Firearm-related deaths remain a significant global public health and medicolegal concern, with marked regional variation in their epidemiological and forensic characteristics. Autopsy-based studies provide essential data for understanding injury patterns and mechanisms of death.

**Objective:**

This study aimed to evaluate the demographic characteristics, injury patterns, shooting distance findings, and toxicological features of firearm-related deaths, and to assess their implications for medicolegal practice.

**Materials and methods:**

A retrospective analysis was conducted on 325 firearm-related deaths identified among 7,139 autopsies performed between 2019 and 2023 at the Council of Forensic Medicine, Bursa Group Presidency. Data were obtained from autopsy reports and archival records. Variables included demographic features, firearm type, shooting distance, entrance wound characteristics, injury localization, associated trauma, and toxicological findings.

**Results:**

Firearm-related deaths accounted for 4.55% of all autopsies. Most cases were male (87%), with a mean age of 36.6 ± 15.3 years. Handguns were used in 94.2% of cases. Contact-range shootings were most frequent (45.5%); however, shooting distance could not be determined in a substantial proportion of cases due to clothing interference or medical intervention. Single entrance wounds were observed in 68.9% of cases, most commonly involving the head (61.2%) and thorax (39.1%). Major vascular injury and bone fractures were present in 22.8 and 89.8% of cases, respectively. Toxicological analysis revealed alcohol in 20.3%, stimulants in 12.6%, and illicit drugs in 6.2% of cases. Some cases demonstrated simultaneous positivity for more than one psychoactive substance; therefore, overlap between stimulant and illicit drug categories was possible.

**Conclusion:**

Firearm-related deaths predominantly affect young adult males and are associated with high lethality due to injuries involving vital anatomical regions. Autopsy findings alone may be insufficient for determining shooting distance. Integration of autopsy findings with scene investigation, clothing analysis, and clinical data is essential for accurate medicolegal interpretation.

## Introduction

Firearm-related injuries result in more than 200,000 deaths annually worldwide, with a substantial proportion attributable to homicide, suicide, and accidental events (1–4). In Türkiye, firearm injuries continue to represent a significant public health concern in terms of both mortality and morbidity, with thousands of injuries and deaths reported each year (5).

The frequency of deaths due to firearm injuries, as well as their demographic characteristics, types of firearms used, and injury patterns, vary considerably depending on regional and sociocultural factors. Therefore, data obtained from different geographical regions are essential for understanding the epidemiological and medicolegal characteristics of firearm-related deaths (6, 7).

In the medicolegal evaluation of firearm-related injuries, parameters such as shooting distance, shooting direction, number of entrance wounds, injury patterns, and type of firearm used are of critical importance for event reconstruction and determination of the cause of death. In such cases, the combined assessment of autopsy findings with scene investigation data, judicial investigation records, and medical documentation constitutes the fundamental approach for establishing the causal relationship between injury and death (8).

Firearm-related injuries continue to represent a major health and safety concern at both global and national levels, and the frequency and characteristics of these deaths vary across populations (3, 5, 9–11). In this context, autopsy-based studies constitute an important source of data for elucidating the demographic and medicolegal characteristics of deaths due to firearm injuries.

The aim of this study was to retrospectively evaluate the demographic characteristics, injury patterns, shooting distance findings, and toxicological features of firearm-related deaths autopsied between 2019 and 2023 at the Bursa Group Presidency of the Council of Forensic Medicine. Furthermore, the study aimed to assess the contribution and limitations of these findings in the medicolegal evaluation of firearm-related injuries.

## Materials and methods

This retrospective study was conducted following approval from the Balıkesir University Faculty of Medicine Health Sciences Ethics Committee (Date: 22 March 2024; Decision no: E-11811414-050.04-366306) and with official permission from the Presidency of the Council of Forensic Medicine, Ministry of Justice of the Republic of Türkiye (Date: 02 July 2024; no: 21589509/2024/678).

Within the scope of the study, archival records of a total of 7,139 autopsy cases performed between 2019 and 2023 at the Morgue Department of the Bursa Group Presidency of the Council of Forensic Medicine were retrospectively reviewed. Among these autopsies, a total of 326 cases involving firearm-related injuries were identified.

Cases in which advanced decomposition prevented reliable evaluation of essential forensic parameters, including shooting distance determination, entrance and exit wound assessment, injury localization, and toxicological analysis, were defined as exclusion criteria. Accordingly, one severely decomposed case was excluded because adequate forensic evaluation could not be performed, and the final study population consisted of 325 cases.

In all included cases, firearm injury was determined to be a direct or major contributing cause of death according to forensic autopsy reports. No cases with uncertain contribution of firearm injury to death were identified. Cases involving combined trauma mechanisms were retained in the analysis when firearm injury itself was independently fatal or represented a major component of the lethal process.

For the purposes of the present study, “firearm-related death” was defined as any case in which firearm injury was identified as the direct cause of death or as an independently fatal injury sufficient to explain death according to forensic autopsy findings. Cases involving combined trauma mechanisms were included only when firearm-related injury alone demonstrated sufficient severity to independently explain death. Data were obtained from autopsy reports and relevant archival records.

Toxicological examinations were performed on biological samples obtained during forensic autopsies. Blood, urine, and vitreous humor samples were routinely collected in all cases. In addition, gastric contents, bile, liver tissue, kidney tissue, nasal swab samples, and adipose tissue specimens were collected for toxicological evaluation when deemed appropriate by the forensic specialist according to the characteristics of the case and autopsy findings.

Systematic toxicological screening analyses for drugs, illicit substances, stimulants, and other toxic agents were conducted using LC–MS/MS, QTOF, and GC–MS platforms. Blood alcohol analyses were performed using HS/GC systems, while volatile substances were analyzed using HS/GC–MS methods.

Each case was evaluated in terms of age, sex, time of the incident, place of death, type of firearm used, shooting distance findings, number of entrance wounds, injury localization, injured organs, associated traumatic findings, and toxicological analysis results.

Gunshot trajectories were considered independently fatal when they resulted in major vascular injury, severe intracranial destruction, or extensive destruction of vital internal organs sufficient to explain death independently according to forensic autopsy evaluation. Bone fractures, projectile localization, and projectile trajectory characteristics were evaluated using both radiological and macroscopic forensic autopsy findings. In firearm-related deaths, routine radiological examinations (X-ray imaging, etc.) were performed to identify retained projectile fragments, evaluate skeletal injuries, and determine projectile direction within the body. In addition, bone fractures and organ injuries were assessed macroscopically during autopsy examination and documented photographically. Fracture findings were further supported by external examination findings such as angulation, deformity, and crepitation detected during routine external examination of each case.

Shooting distance was assessed based on macroscopic findings observed during autopsy, including soot deposition, gunpowder residues, burning, and tattooing. Cases were classified as contact, near-contact, intermediate-range, or distant-range shootings. In cases where the bullet passed through clothing, shooting distance could not be directly determined and such cases were categorized as requiring further examination. In addition, cases in which sufficient findings could not be obtained due to medical intervention or postmortem changes were recorded as indeterminate.

Statistical analyses were performed using SPSS version 26.0 (IBM Corp., Armonk, NY, United States). In descriptive statistics, continuous variables were expressed as mean ± standard deviation or median (minimum–maximum), while categorical variables were presented as counts and percentages (%). The chi-square test was used for comparisons of categorical variables, including the evaluation of seasonal differences between case frequencies. Annual comparisons of firearm-related deaths were performed using proportional distributions relative to the total number of autopsies for each year rather than absolute case counts alone. Continuous variables were analyzed using parametric or non-parametric methods according to data distribution characteristics. Comparisons of age distributions between male and female cases were performed using the Mann–Whitney U test. Additional subgroup analyses between injury localization, shooting distance, and place of death were not performed because the retrospective archival structure of the dataset did not allow reliable reconstruction of the original shooting scene in a substantial proportion of hospital-based deaths.

Due to the retrospective design of the study and the reliance on autopsy reports and archival records, full access to scene investigation findings, judicial case files, and court decisions was not available. This constitutes a limitation, particularly with regard to the assessment of shooting distance and the scope of medicolegal interpretation.

Although the study was based on autopsy cases evaluated at a single regional forensic center, Bursa is one of the largest metropolitan regions in Türkiye and the Bursa Group Presidency of the Council of Forensic Medicine also receives medicolegal cases from several surrounding provinces. Therefore, the study population reflects a relatively broad regional forensic spectrum despite the retrospective single-center design.

## Results

**Figure 1:**
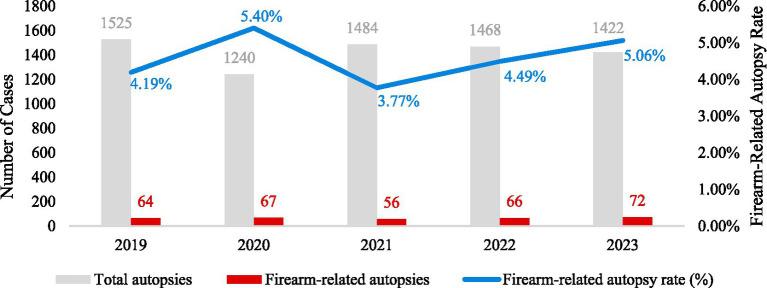
Annual distribution of total autopsies, firearm-related autopsies, and the proportion of firearm-related cases between 2019 and 2023.

Of the 7,139 autopsies performed between 2019 and 2023 at the Morgue Department of the Bursa Group Presidency of the Council of Forensic Medicine, 325 cases (4.55%) were attributed to firearm-related deaths. When the annual distribution was examined, the highest absolute number of firearm-related autopsy cases was observed in 2023, whereas the highest proportion of firearm-related deaths relative to the total number of autopsies was observed in 2020. Comparisons between years were performed based on proportional distributions relative to total annual autopsy numbers using chi-square analysis, and no statistically significant difference was identified (*p* > 0.05) (Figure 1).

The majority of cases were male (87%, *n* = 282), while females accounted for 13% (*n* = 43). The mean age was 36.63 ± 15.28 years (range: 5–96), with the highest proportion of cases observed in the 31–40 age group (26.15%, *n* = 85). The mean ages of male and female cases were 40.07 ± 15.70 and 36.74 ± 11.88 years, respectively, and no statistically significant difference was identified between the sexes according to Mann–Whitney U analysis (*p* > 0.05).

Cases under 18 years of age accounted for 8 (2.5%) of the total, including 6 males and 2 females.

Overall, an increase in cases was noted during the summer and winter months, whereas a relatively lower frequency was observed in spring and autumn. This seasonal variation was found to be statistically significant according to chi-square analysis (*p* < 0.05).

When the place of death was examined, 41.2% of cases (*n* = 134) occurred in hospital settings, while 24.0% (*n* = 78) occurred at home (Table 1).

**Table 1:** Demographic characteristics and temporal patterns of firearm-related deaths (*n* = 325).

	*n*	%
Age group (years)		
0–10	4	1.2
11–20	19	5.8
21–30	75	23.1
31–40	85	26.1
41–50	65	20.0
51–60	44	13.5
61–70	22	6.8
71–80	8	2.5
>80	3	0.9
Sex		
Male	282	87
Female	43	13
Place of death		
Hospital	134	41.2
Home	78	24.0
Public place	58	17.8
Workplace	19	5.9
Vehicle	10	3.1
Unknown	26	8.0
Season of death		
Spring	66	20.3
Summer	106	32.6
Autumn	68	20.9
Winter	85	26.2
Day of the week		
Weekdays	234	72.0
Weekends	91	28.0

Regarding the number of entrance wounds, a single entrance wound was observed in 68.9% of cases (*n* = 224), whereas multiple entrance wounds were present in 31.1% (*n* = 101). Among cases with multiple entrance wounds, two entrance wounds were the most common pattern (*n* = 28). In rare instances, cases involving as many as 13 and 23 entrance wounds were also identified. In approximately 20% of cases with multiple entrance wounds, all gunshot trajectories were considered independently fatal based on the presence of major vascular injury, severe intracranial destruction, or extensive injury to vital internal organs sufficient to explain death independently. When firearm types were evaluated, handguns were involved in the vast majority of cases (94.2%, *n* = 306), followed by shotguns in 5.5% (*n* = 18) and the combined use of both firearm types in 0.3% (*n* = 1).

Among cases in which shooting distance could be determined, contact-range shootings represented the most frequent category (*n* = 148). However, in a substantial proportion of cases, shooting distance could not be reliably determined because of clothing interference, medical intervention, or postmortem decomposition, and these cases were categorized as indeterminate (Tables 2, 3).

**Table 2:** Characteristics of firearm injuries: shooting distance, entrance wound count, and anatomical distribution (*n* = 325).

	*n*	%
Shooting distance		
Contact	148	45.5
Near-contact	5	1.5
Intermediate range	12	3.7
Distant	46	14.2
Indeterminate*	154	47.4
Number of entrance wounds		
1	224	68.9
2	28	8.6
3	17	5.2
4	19	5.8
5	13	4.0
≥6	24	7.4
Anatomical distribution**		
Head	199	61.2
Thorax	127	39.1
Abdomen	83	25.5
Upper extremities	47	14.5
Lower extremities	42	12.9
Neck	32	9.8
Major vascular injury		
Yes	74	22.8
No	251	77.2
Bone fracture		
Yes	292	89.8
No	33	10.2

*Indeterminate cases do not represent a true shooting-distance category but rather cases in which reliable shooting-distance assessment could not be performed because of clothing interference, medical intervention, decomposition, or insufficient wound characteristics. **Percentages for anatomical injury distribution may exceed 100% because multiple anatomical regions could be affected in the same case.

**Table 3:** Distribution of skeletal injuries in firearm-related deaths (*n* = 325).

	*n*	%
Skull	187	57.5
Ribs	99	30.5
Vertebrae	41	12.6
Face	26	8.0
Upper extremities	19	5.8
Sternum	17	5.2
Lower extremities	16	4.9
Pelvis	14	4.3
Scapula	6	1.8

When internal organ injuries were evaluated, the central nervous system (brain and cerebellum) was the most frequently affected structure (58.8%, *n* = 191), followed by the lungs, heart, and intestines. The least frequently injured organs were the genital organs (Table 4).

**Table 4:** Distribution of injured internal organs in firearm-related deaths (*n* = 325).

	*n*	%
Central nervous system		
Brain	191	58.8
Spinal cord	8	2.5
Thoracic organs		
Lungs	152	46.8
Heart	69	21.2
Trachea and esophagus	11	3.4
Abdominal organs		
Intestines	48	14.8
Liver	40	12.3
Stomach	26	8.0
Kidneys	20	6.2
Spleen	17	5.2
Other organs		
Diaphragm	34	10.5
Genital organs	2	0.6
**No internal organ injury identified**	**5**	1.5

Bold values indicate the main anatomical/system categories used for grouping the injured organs.

In 61.2% of cases (*n* = 199), death was attributed to injury of a single organ. In the majority of these cases, brain injury predominated (*n* = 163), followed less frequently by injuries to the lungs (*n* = 12), intestines (*n* = 12), heart (*n* = 4), esophagus (*n* = 3), spinal cord (*n* = 3), kidneys (*n* = 1), and spleen (*n* = 1).

In six cases, death occurred due to isolated major vascular injury without associated internal organ damage. The affected vessels in these cases included the femoral artery and vein (*n* = 3), tibial artery (*n* = 1), iliac artery and vein (*n* = 1), and the jugular vein and common carotid artery (*n* = 1).

In addition to firearm-related injuries, a small number of cases demonstrated the presence of additional traumatic mechanisms. These included blunt force trauma in 2 cases, combined sharp and blunt force injury in 1 case, hanging in 1 case, and drowning in 1 case.

**Figure 2:**
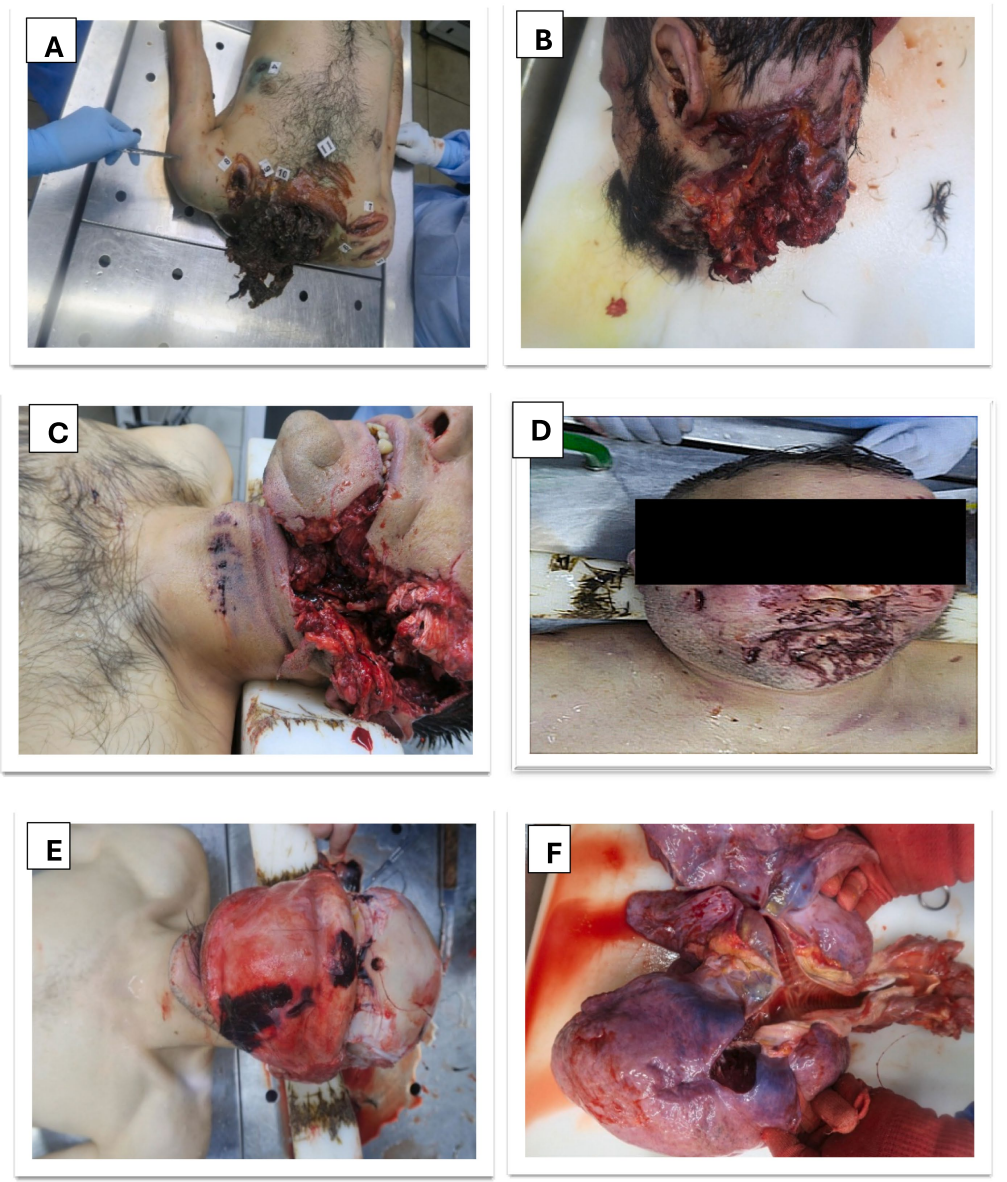
Representative autopsy findings demonstrating complex firearm-related injury patterns and combined trauma mechanisms. **(A,B)** Firearm injury associated with postmortem sharp-force and blunt trauma findings. **(C)** Firearm injury associated with antemortem hanging findings. **(D)** Firearm injury associated with antemortem blunt-force trauma. **(E,F)** Firearm injury associated with antemortem drowning findings.

It was determined that the sharp and blunt force injury occurred after death due to firearm injury, whereas the additional injuries in the remaining cases were sustained during the antemortem period and were of a nature that could contribute to death in conjunction with the firearm injury (see Figure 2).

In toxicological analyses, alcohol was detected in 20.3% of cases (*n* = 66), stimulants in 12.6% (*n* = 41), and illicit drugs in 6.2% (*n* = 20).

Among cases with detected illicit drugs, the majority involved the tetrahydrocannabinol (THC) metabolite THC-COOH (*n* = 18), while synthetic cannabinoid derivatives (ADB-BUTINACA, MDMB-4en-PINACA, 5F-ADB) were identified in a smaller number of cases.

Among cases with detected stimulants, most involved amphetamine and/or methamphetamine (*n* = 38), followed less frequently by cocaine metabolites (benzoylecgonine, methylecgonine, ecgonine; *n* = 7) and other amphetamine derivatives (norpseudoephedrine, ephedrine, pseudoephedrine; *n* = 6). In one case, MDMA (3,4-methylenedioxymethamphetamine) was detected.

## Discussion

In the present study, firearm-related deaths accounted for 4.55% of all medicolegal autopsies. Previous studies conducted in different regions of Türkiye have reported rates ranging from 4.2 to 34.7% (6, 7, 12–18). Similarly, international studies have demonstrated that the proportion of firearm-related deaths varies between 1.5 and 29% (19–22). These findings indicate that the frequency of firearm-related deaths is substantially influenced by regional and structural factors, including access to firearms, patterns of use, and legal regulations (4). In this context, autopsy-based studies represent an important source of data for identifying regional differences in firearm-related mortality.

The predominance of male cases (87%) in our study reflects a marked male predominance in firearm-related deaths. In the literature, the proportion of male cases has been reported to range between 78 and 94% (4, 6, 12–14, 17, 18, 21–23), indicating that our findings are consistent with previous reports. The mean age of cases was 36.6 ± 15.3 years, with the majority (69%) falling within the 21–50 age group. This age group has also been identified as the most affected population in previous studies (6, 23, 24). This demographic pattern suggests that firearm-related deaths predominantly affect individuals in their most active period of life and, from a medicolegal perspective, may represent a population at higher risk in terms of both victimization and involvement in such events.

In our study, the proportion of firearm-related deaths among individuals under 18 years of age was 2.5%. Although this rate is lower compared to higher proportions reported in some regions of Türkiye (7, 25), it nevertheless indicates that firearm-related injuries remain an important cause of mortality in the pediatric and adolescent population. The persistence of male predominance in this age group suggests that gender-related risk factors associated with firearm injuries may be effective from early ages (7, 25, 26). These findings highlight the importance of firearm accessibility, supervision, and safe storage practices in pediatric populations.

When the temporal distribution of cases was examined, the highest number of cases occurred during the summer months, followed by winter. However, previous national and international studies have reported heterogeneous findings regarding seasonal variation (15, 16). Therefore, it is difficult to establish a consistent seasonal pattern for firearm-related deaths, and the observed differences are likely to be influenced by regional and environmental factors.

When the place of death was evaluated, 41% of cases occurred in hospital settings, 24% at home, and 18% in public places, while in 8% of cases the place of death could not be determined. These findings indicate that firearm-related deaths should be evaluated by considering both deaths occurring at the scene and those occurring after hospital admission. Previous studies have also reported variability in the distribution of place of death across different centers (13, 27). These differences may be associated with factors such as injury severity, scene conditions, and access to emergency medical services. These findings further emphasize the importance of integrating scene investigation findings with autopsy data during medicolegal evaluation.

When firearm type was examined, short-barreled firearms were used in the vast majority of cases (94.2%), while long-barreled firearms were used less frequently (5.5%). The distribution of firearm types has also been reported to vary across regions in the literature (6, 13, 16, 17). The predominance of short-barreled firearms may be related to their portability and accessibility. However, it is clear that the effect of firearm type on injury patterns, shooting distance, and the mechanism of death should be evaluated on a case-by-case basis.

Beyond firearm accessibility and portability, the high proportion of contact-range shootings may also reflect the close interpersonal nature of many firearm-related fatal events discussed in the forensic literature, including domestic violence incidents, interpersonal assaults, organized criminal activity, and self-inflicted firearm injuries. However, because the present study did not include definitive medicolegal classification of manner of death, these interpretations should be approached cautiously within the limitations of retrospective autopsy-based evaluation. In our study, analysis of shooting distance distribution revealed that contact-range shootings were the most frequent (45.5%), followed by near-contact (1.5%), intermediate-range (3.7%), and distant-range shootings (14.2%). However, in a substantial proportion of cases, shooting distance could not be determined during autopsy due to clothing interference (41.9%) and loss of relevant findings during medical intervention (4.9%). These findings clearly demonstrate that shooting distance cannot always be reliably determined based solely on autopsy findings in firearm-related injuries.

Previous studies have reported considerable variability in shooting distance distribution, with some series identifying contact-range shootings as predominant, while others report a higher frequency of distant-range shootings (6, 16–18). These differences may be associated with variations in case origin, types of firearms used, and, in particular, the masking effects of clothing as well as alterations in wound characteristics following medical intervention.

From a medicolegal perspective, clothing interference and alterations in wound characteristics caused by medical intervention may significantly limit reliable determination of shooting distance. Therefore, the integration of scene investigation data, clothing examination, and clinical documentation is essential for a more reliable determination of shooting distance.

When the number of entrance wounds was evaluated, a single entrance wound was identified in 68.9% of cases, whereas multiple entrance wounds were present in 31.1%. Among cases with multiple entrance wounds, all gunshots were determined to be fatal in 20% of cases. Previous studies have reported single entrance wound rates ranging between 60 and 70% (16, 17), indicating that our findings are consistent with the literature. The predominance of single entrance wounds underscores the importance of injury patterns involving vital organs with high lethality in firearm-related injuries.

Although the number of entrance wounds represents an important parameter in the medicolegal evaluation of firearm-related injuries, it is not sufficient on its own to determine the manner of the event. Therefore, the number of entrance wounds should be interpreted in conjunction with scene findings and other forensic evidence (28, 29). Cases involving multiple entrance wounds require more complex medicolegal evaluation regarding event dynamics and injury patterns. When injury localization was evaluated, the head (61.2%) and thoracic region (39.1%) were the most frequently affected sites, followed by the abdominal region (25.5%) and the extremities. Similarly, previous studies have reported that regions containing vital organs, particularly the head–neck and thoracic regions, are most commonly involved (16, 18).

Injuries involving the head and thorax are known to be associated with high lethality, emphasizing the critical role of injury localization in firearm-related deaths.

These findings further demonstrate that, in firearm-related injuries, targeting vital anatomical regions may result in high lethality even with a limited number of shots, and that fatal outcomes may occur independently of the number of entrance wounds.

In our study, toxicological analyses revealed alcohol in 20.3% of cases, stimulants in 12.6%, and illicit drugs in 6.2%. Previous studies have reported variable rates of alcohol and psychoactive substance use in firearm-related deaths (30, 31). Toxicological findings in the present study were evaluated descriptively and were not subjected to additional inferential correlation analyses regarding shooting distance, injury localization, or event dynamics. Therefore, these findings should be interpreted cautiously within the limitations of the retrospective study design. In some cases, more than one psychoactive substance was detected simultaneously; accordingly, overlap between stimulant and illicit drug categories was possible.

In addition, a small number of cases in our study demonstrated the presence of accompanying injuries alongside firearm-related trauma, including blunt force trauma, combined sharp and blunt force injury, as well as distinct mechanisms such as hanging and drowning. Previous reports have also described rare cases in which firearm injuries are associated with multiple lethal mechanisms, complicating medicolegal evaluation (32–34).

In such cases, the coexistence of multiple mechanisms of death necessitates a comprehensive and multidimensional evaluation to accurately determine both the dynamics of the event and the manner of death. In this context, the combined assessment of scene findings, autopsy results, and toxicological data is of critical importance. Furthermore, distinguishing whether associated injuries occurred during the antemortem or postmortem period represents a key step in the medicolegal evaluation process.

In the present study, no analysis was performed regarding the manner of death. The primary reason for this was the lack of full access to prosecutorial files, law enforcement records, and court decisions within the retrospective archival data, as well as the fact that autopsy findings alone do not always provide sufficient information for determining the manner of death. Therefore, the integration of scene investigation findings, judicial records, and clinical documentation is essential.

Several retrospective studies in the forensic literature have attempted to infer the manner of death primarily from autopsy findings and wound characteristics. However, in contemporary forensic practice, definitive classification of firearm-related deaths as suicide, homicide, or accident requires the integrated evaluation of scene investigation findings, witness statements, judicial records, behavioral background, and final court decisions in addition to autopsy data. Considering the retrospective design of the present study and the limited access to active prosecutorial and court files from different judicial districts, the authors deliberately avoided retrospective categorization of cases based solely on autopsy findings in order to minimize potential misclassification bias.

This study has several limitations. A major limitation of the present study is the absence of definitive manner-of-death classification, including suicide, homicide, accidental, and undetermined categories. Although many firearm autopsy series include such classifications, the authors deliberately avoided retrospective manner-of-death categorization based solely on autopsy findings because medicolegal determination of manner of death often requires comprehensive integration of autopsy findings with scene investigation, witness statements, ballistic evaluation, prosecutorial investigation, and final judicial decisions. In many cases, these judicial and legal processes may remain ongoing for prolonged periods and may subsequently lead to changes in the final legal classification of death. Therefore, retrospective classification based on incomplete medicolegal data could increase the risk of misclassification bias. Nevertheless, the absence of manner-of-death analysis reduces the interpretive scope of the study and limits direct comparison with previously published firearm autopsy series.

Therefore, the findings of this study should be interpreted within the context of the analyzed autopsy series, and caution should be exercised when generalizing the results to broader populations. Nevertheless, the relatively large autopsy dataset constitutes a strength of this study and provides valuable contributions to the medicolegal evaluation of firearm-related injuries.

## Conclusion

This study presents a large autopsy-based series evaluating the demographic distribution, injury patterns, and medicolegal characteristics of firearm-related deaths. The findings demonstrate that such deaths predominantly affect males in their most active period of life and constitute a significant proportion of medicolegal autopsies.

Analysis of injury patterns revealed a predominance of head and thoracic injuries, with single entrance wounds frequently resulting in fatal outcomes. These findings indicate that targeting vital anatomical regions is strongly associated with high lethality in firearm-related injuries.

The findings emphasize the importance of integrating autopsy findings with scene investigation, clothing examination, and clinical documentation for accurate medicolegal interpretation. The present study contributes regional forensic epidemiological data and highlights important practical considerations in the medicolegal evaluation of firearm-related deaths. These findings have direct implications for the reconstruction of firearm-related events in medicolegal investigations.

## Data Availability

The datasets generated and/or analyzed during the current study are not publicly available because they contain official forensic case records obtained with institutional permission from the Council of Forensic Medicine, Ministry of Justice of the Republic of Türkiye. Access to these data may be considered only upon official application and with approval from the relevant institutional authorities.
